# Does simultaneous soft tissue augmentation around immediate or delayed dental implant placement using sub-epithelial connective tissue graft provide better outcomes compared to other treatment options? A systematic review and meta-analysis

**DOI:** 10.1371/journal.pone.0261513

**Published:** 2022-02-10

**Authors:** Taghrid Aldhohrah, Ge Qin, Dongliang Liang, Wanxing Song, Linhu Ge, Mubarak Ahmed Mashrah, Liping Wang

**Affiliations:** Department of dental implantology, Affiliated Stomatology Hospital of Guangzhou Medical University, Guangdong Engineering Research Center of Oral Restoration and Reconstruction, Guangzhou Key Laboratory of Basic and Applied Research of Oral Regenerative Medicine, Guangzhou, Guangdong, China; Thamar University, Faculty of Dentistry, YEMEN

## Abstract

**Objective:**

The clinical benefits of simultaneous implant placement and soft tissue augmentation using different treatment modalities are unclear. The current meta-analysis aimed to compare the effect of simultaneous soft tissue augmentation using subepithelial connective tissue graft (SCTG) around immediate or delayed dental implant placement with other treatment modalities on the peri-implant tissue health and esthetic.

**Methods:**

Up to May 2021, four databases (PubMed, EMBASE, Cochrane Central, and Google Scholar) were searched. Randomized control trials with follow-up >3 months, evaluating simultaneous implant placement (immediate or delayed) and soft tissue augmentation using SCTG compared with other treatment modalities were included. The predictor variables were SCTG versus no augmentation with/without guided bone regeneration (GBR) or other augmentation techniques (Acellular dermal matrix (ADM), Xenogeneic collagen matrix (XCM). The outcome variables were buccal tissue thickness (BTT), mid-buccal gingival level (MGL), marginal bone loss (MBL), and pink esthetic scores (PES). Cumulative mean differences (MD) and 95% confidence interval (CI) were estimated.

**Results:**

Twelve studies were included. SCTG along with immediate implant placement (IIP) or delayed implant placement (DIP) showed a statistically significant improvement in BTT (Fixed; MD, 0.74; 95% CI, 0.51; 0.97), MGL (Fixed; MD, 0.5; 95% CI, 0.21; 0.80), PES (Fixed; MD, 0.79; 95% CI, 0.29; 1.29), and less MBL (Fixed; MD, -0.11; 95% CI, -0.14; -0.08) compared to no graft (P<0.05). A statistically insignificant differences in BTT (Random; MD, 0.62; 95% CI, -0.41; 1.65), MGL (Fixed; MD, -0.06; 95% CI, -0.23; 0.11), MBL (Fixed; MD, 0.36; 95% CI, -0.05; 0.77) and PES (Fixed; MD, 0.28; 95% CI, -0.10; 0.67) was observed when SCTG along with DIP was compared with no augmentation plus GBR. Similarly, no statistically significant difference was observed when comparing SCTG along with DIP with acellular dermal matrix (ADM) concerning BTT (MD:0.71, P = 0.18) and KMW (MD: 0.6, P = 0.19).

**Conclusion:**

There is a very low quality of evidence to provide recommendations on whether simultaneous dental implant placement (IIP or DIP) and soft tissue augmentation using SCTG is superior to no augmentation or is comparable to the other tissue augmentation materials in improving the quality and quantity of peri-implant tissues. Therefore, further, well-designed RCTs with larger sample sizes and long follow-up times are still needed.

## 1. Introduction

Dental implants are widely used for the replacement of missing teeth. Recently, osseointegration around dental implants comes to be a foreseeable procedure; therefore, the focus has been shifted from obtaining osseointegration to achieve a satisfying aesthetic appearance [1, 2]. Providing a naturally looking peri-implant tissue, particularly in the esthetic zone, is a complex and challenging undertaking for the dental implant team. Adequate buccolingual and apicocoronal dimensions of hard and soft tissues are essential for optimal function and esthetic after dental implantation [3, 4]. Sometimes the placement of the dental implant in the esthetic zone either into healed bone or into the extraction socket is associated with esthetic problems especially for patients who show their maxillary gingival scallop while smiling or talking [5]. Esthetics complications are usually caused by a lack of sufficient bone after tooth loss. Management of bone deficiency prior to or at the time of dental implant placement using several bone augmentation techniques has been summarized in Cochrane Systemic reviews [6, 7]. However, there are situations in which it might be possible to solve the unpleasant esthetic results solely through manipulating or augmenting soft tissues [8]. Soft tissue augmentation can be carried out at different time points during implant treatment either simultaneously, during the phase of tissue integration or it can be delayed after final implant loading [2]. Simultaneous soft tissue augmentation at the time of dental implant placement using subepithelial connective tissue graft (SCTG) [9] or other substitutes such as xenogenic collagen matrix (XCM) [10, 11], acellular dermal matrix (ADM) [12] has been recommended to reduce crestal bone loss in a patient with thin gingival biotype [13, 14], to prevent mid-facial mucosal recession [14, 15], to avoid shimmering through implant parts, especially those made of titanium [16].

SCTG harvested from the hard palate or tuberosity region has become the gold standard technique to thicken peri-implant tissue and to improve esthetic. However, SCTG has been criticized to be associated with donor site morbidity and long operative time. To overcome such downsides, XCM and ADM have been used as an alternative to the SCTG for soft tissue augmentation around the dental implant. Recently, a considerable number of systemic reviews and meta-analyses concerned with the effectiveness of soft tissue augmentation in the healthy and diseased soft tissue around dental implant, [17] timing of graft placement [2], the changes of keratinized thickness [18], and the effect of augmentation on the esthetic outcomes around dental implant [19] or evaluate success rate, and complications associated between type 1 and other types of implant placement protocols [20] have been published. Lin et al [2] showed that no difference between simultaneous and staged soft tissue augmentation during implant treatment. Thoma et al [17] concluded that soft tissue grafting procedures result in more favorable peri-implant KMW, BTT, and MBL, compared to no grafting protocol. In another systematic review, Esposito et al [8] concluded that there is insufficient reliable evidence to provide recommendations on whether techniques to correct/augment peri implant soft tissues or to increase the width of keratinized/attached mucosa are beneficial to patients or not.

Recently, Stefan, et al conducted a systematic review and they reported that soft tissue augmentation is beneficial regarding width of keratinized mucosa and midfacial recession and showed no influence regarding peri implant MBL [21]. Similarly, Angelis et al found that SCTG improve peri implant soft tissue thickness and alleviate soft tissue recession and marginal bone loss when placed simultaneously with IIP protocol [22]. However, there is a lack of clear evidence regarding the clinical and aesthetic benefits of simultaneous soft tissue augmentation around immediate or delayed dental implant placement using SCTG compared with no grafting (with or without GBR) or with different augmentation procedures (CM and ADM). Therefore, this study was conducted to systemically review and critically evaluate studies that compared soft tissue changes after various augmentation techniques at the dental implant site and to answer the question “Does simultaneous soft tissue augmentation around immediate or delayed dental implant placement using SCTG provide better outcomes compared to other treatment options?”.

Changes in the buccal soft tissue thickness buccal (BTT), mid-buccal gingival level (MGL), marginal bone loss (MBL), keratinized tissue width (KMW), and Pink esthetic score (PES) were considered as the predictors of comparisons between different surgical procedures.

## 2. Materials and methods

In this systematic review and meta-analyses, the authors follow the Preferred Reporting Items for Systematic Review and Meta-analyses (PRISMA) statement (S1 Checklist) [23]. The protocol of this meta-analysis has been registered in PROSPERO (registration number CRD42019123118).

### 2.1 Focused question

Does simultaneous soft tissue augmentation at the time of immediate or delayed implant placement using subepithelial connective tissue graft provide better outcomes compared to other treatment options?

The question for the current meta-analysis was adopted to follow PICO criteria:

P: Adult healthy partially edentulous patients received single dental implant placement in the extraction socket or healed site.
I: Soft tissue augmentation using SCTG (harvested from the palate or maxillary tuberosity) or other augmentation materials (ADM, or XCM) around immediate or delayed dental implant placement.
C: SCTG, No augmentation or other augmentation materials (ADM, or XCM).
O: Change in the BTT, MGL, MBL, KMW, and PES, in >3 months follow-up period.
**T**: The patients in all included studies should be followed for more than 3 months after simultaneous implant placement and soft tissue augmentation.
**S**: Randomized controlled trials (RCTs) (split-mouth and parallel studies).

Change in MGL is defined as apical migration of the gingival margin toward the platform of the dental implant. BTT is measured 1 to 2 mm below the implant gingival margin and classified as thin gingival biotype (if ≤ 1 mm) or thick gingival biotype (if > 1 mm). MBL is defined as the distance from the implant-abutment interface on the implant side to the marginal bone. KMW is defined as the distance between the gingival margin and the mucogingival junction.

### 2.2 Search strategy

From inception to May 2021, An electronic search of PubMed, EMBASE, and Cochrane Central, Google Scholar was performed by two reviewers independently (S1 File). Incorporation of the following keywords were used for the electronic search in PubMed: ((immediate implant [Title/Abstract]) OR (immediate implant placement [Title/Abstract]) OR (early implant placement [Title/Abstract])) OR (delayed implant placement [Title/Abstract])) AND ((soft tissue graft [Title/Abstract]) OR (sub-epithelial connective tissue graft [Title/Abstract]) OR (connective tissue [Title/Abstract]) OR (soft tissue augmentation [Title/Abstract]) OR (soft tissue transplantation [Title/Abstract]) OR Xenograft [Title/Abstract])) OR heterografts [Title/Abstract])) OR collagen matrix [Title/Abstract])) OR mucograft [Title/Abstract])) OR Acellular dermal matrix [Title/Abstract])) OR acellular dermis [Title/Abstract])) AND ((attached gingiva[Title/Abstract]) OR (buccal soft tissue thickness [Title/Abstract]) OR (keratinized mucosa[Title/Abstract]) OR (soft tissue margin[Title/Abstract]) OR (pocket probing depth [Title/Abstract]) OR (esthetic [Title/Abstract])). Besides, a manual searching in the field of dental implantology (e.g. Clinical Oral Implants Research, Journal of dentistry, Clinical Oral Implant Dentistry and Related Research, Journal of Periodontology research, Journal of Clinical Periodontology, Journal of Periodontology, Journal of Oral Rehabilitation, International Journal of Oral and Maxillofacial Implantology, International Journal of Oral and Maxillofacial Surgery, American Journal of oral and maxillofacial surgery and European Journal of Oral Implantology, Journal of Esthetic and Restorative Dentistry) was also carried out. The related articles were carefully checked for studies that met the inclusion criteria.

### 2.3 Inclusion/exclusion criteria

Qualified studies that fulfill the following criteria were included: 1) English-language human randomized controlled trials (RCTs), 2) Single dental implant placed in the extraction socket or healed site with simultaneous soft tissue augmentation 3) RCTs with follow-up >3months. 4) RCTs that compared SCTG with other augmentation techniques. 5) RCTs that reported at least one of the following variables: BTT, KMW, MGL, MBL, or PES.

Studies that reported one of the following criteria were excluded: 1) not RCT and no simultaneous soft tissue augmentation was performed at the time of dental implant placement. 2) Sample size less than 10 patients, 3) review studies, meeting abstracts, case reports, case series, and non-English articles. 3) Studies < 3month follow-up period.

### 2.4 Data extraction process

Two researchers (TA., GQ.) independently assessed the titles, abstracts, and full-text of the relevant studies. All of the following data in the included studies were collected when available: study design, number of patients, publication year, age range, mean age, implant number, company, type of intervention, flap or flapless, hard tissue augmentation, follow-up period, and outcome variables (Table 1). Two researchers (A. TA. DLL.) collected the data regarding outcomes of interest, any disagreements between the reviewers were resolved by consensus.

**Table 1:** Characteristics of the included studies.

Author/ Study design	Patients	Age	Groups	Implants	Main outcomes in (mm)	IIP/ IIPP	bovine bone mineral	Flap design	Follow up	Gingival biotype
**Yoshino et al., 2014** [35] **RCT**	20	52.6 yrs.	SCTG NG	20 10/10	**MBL** SCTG –0.01 (0.27) NG –0.14 (0.53) **MGL** SCTG –0.25 (0.35) NG –0.7 (0.48)	Yes	Yes	Full thickness envelope flap	12 mos.	**SCTG** Thin 0 Thick 10 **NG** Thin 3 Thick 7
**Zuiderveld et al. 2018a** [37] **RCT**	60 58 analyzed (29/29)	45.5 yrs.	SCTG NG	60 30/30	**MGL** SCTG 0.0(0.3) NG 0.0(0.3) **MBL S**CTG mesial 0.9 (0.4–1.2), distal 0.8 (0.0–1.1) NG mesial 0.8 (0.5–1.2), distal 0.8 (0.0–1.1) **PES** SCTG 6.4 (1.5) NG 6.8 (1.5) **Probing depth** SCTG mesial 2.8 (1.1), buccal 2.3 (0.9), distal 2.9 (0.9), palatal 2.2 (0.7) NG mesial 3.0 (0.9),buccal 2.5 (1.2), Distal 2.9 (1.4), Palatal 2.3 (0.8)	Yes /No	Yes	Supra-periosteal envelope flap	12 mos.	**SCTG** Thin 20 Thick 10 **NG** Thin 15 Thick 15
**Zuiderveld et al. 2018b** [33] **RCT**	60	47.5	SCTG NG XCM	60 20/20/20	**MGL** SCTG -0.03(0.2) NG -0.15(0.2) XCM -0.16(0.2) **MBL S**CTG mesial 0.3(0.0–0.9), distal 0.5(0.0–1.0) NG mesial 0.5(0.0–0.9) / distal 0.4(0.0–1.1) XCM mesial 0.7(0.3–1.6) / distal 0.6 (0.0–1.1) **PES** SCTG 7.0 (2.4) NG 6.6 (1.5) XCM 6.1(1.7) **Probing depth** SCTG mesial 2.5(1.1), Buccal 2.7 (1.2), Distal 2.3 (0.6), Palatal 2.0 (0.8) NG mesial 2.4(1.3), buccal 3.3(1.2) Distal 2.7(1.1), palatal 2.5(0.7) XCM mesial 2.8 (1.2), buccal 2.8 (1.6) distal 2.9(0.9), Palatal 2.6(0.8)	No	No	Minimal muco-periosteal flap	12 mos.	**SCTG** Thin 13 Thick 7 **NG** Thin 15 Thick 5 **XCM** thin 10 Thick 10
**Migliorati et al. 2013** [36] **RCT**	48 47 analyzed (NG 23)	47.5 yrs.	SCTG NG	48 24 / 24	**Probing depth** SCTG 3.3(0.4) / 3.4(0.5) NG 3.1 (1) / 3.2 (0.5) **Bleeding on probing** SCTG 0.4(0.3) / 0.2(0.3) NG 0.4(0.4) / 0.4(0.4) **MBL** SCTG 0.001(0.092)/ −0.06(0.091) NG −0.136(0.107) / −0.166(0.063) **BTT** SCTG 1.8(0.8) / 1.5(0.8) NG 1.1(0.5) / 1.0 (0.5) **KMW** SCTG 3.0 (1.2) / 2.9 (1.2) NG 3.7 (1.1) / 3.6 (1.2)	Yes /No	Yes	No	24 mos.	SCTG Thin 14 Thick 10 NG Thin 12 Thick 11
**Gretchen A. Wigand 2012** [34] **RCT**	24 22 analyzed (ADM 10)	52 yrs.	SCTG ASDM	24 12 / 12	**MGL** SCTG 0.3 (0.4) ADM 0.5 (0.5) **PES** SCTG 11.6 (1.5) ADM 11.7 (1.6) **Bleeding on Probing** SCTG 0.1(0.1) ADM 0.1 (0.1) **KMW** SCTG 4.7 (0.9) ADM 4.8 (1.1) **Probing depth** SCTG 0.1 (0.1) ADM 1.9 (0.5)	No	No	full thickness mucoperiosteal flap	12 mos.	NR
**Hutton et al 2018** [30] **RCT**	20	55.5 yrs.	SCTG ADM	20 10 / 10	**KMW SCTG** 4.45 (1.14) ADM 4.50 (0.94) **PMT S**CTG 3.61 (1.11) ADM 2.90 (1.24)	No	No	Partial thickness flap	16 weeks	NR
**D’Elia et al., 2017** [31] **RCT**	32 30 analyzed (15/15)	47.5 yrs.	SCTG GBR	32 16/16	**BTT** GBR 3.7 ± 1.1 SCTG 3.73 ±1.13 **MGL** GBR 0.23 ± 0.34 SCTG 0.35 ± 0.56 **Probing depth** GBR 1.9 ± 0.42 SCTG 2.17 ± 0.67 **KMW** GBR 5.16 ± 1.22 SCTG 4.86 ± 0.83	No	-	split-full-split thickness approach	12 mos.	NR
**Wiesner et al, 2010** [5] **RCT**	20	39 yrs.	SCTG NG	40 20/20	**BTT** NG 1.90 (0.32) SCTG 3.20 (0.42) **MBL** SCTG -1.14 (0.29) NG -1.06 (0.41) **PES** SCTG 11.32 (1.63) NG 8.45 (1.46)	No	No	split thickness flap	12 mos.	NR
**De Bruyckere et al. 2018** [28] **RCT**	42	49 yrs.	SCTG GBR	42 21/21	**BTT** GBR 1.59 (0.68) SCTG 2.68 (0.67) **MBL** GBR 0.42 (0.36) SCTG 0.78 (0.88) **Probing depth** GBR 3.27 (0.75) SCTG 3.53 (0.40) **Plaque (%), Bleeding on Probing** GBR 13.16 (17.42), 22.37 (20.23) SCTG 10.71 (20.27), 25.00 (20.92) **MGL** GBR 0.11 (0.39) SCTG 0.24 (0.26)	No	-	Muco-periosteal flap	12 mos.	NR
**De Bruyckere et al. 2020** [32] **RCT**	42 40 analyzed (GBR 19)	49 yrs.	SCTG GBR	42 21/21	**PES** SCTG 10.48 (2.25) GBR 10.11 (1.83) **Mucosal Scarring Index** SCTG 1.10 (1.34) GBR 2.53 (2.12)	No	-	Muco-periosteal flap	12 mos.	NR
**Frizzera et al., 2018** [29] **RCT**	24	65yrs	CTG XCM NG	24 8/8/8	**PES** SCTG 10.75 (1.38) XCM 10 (1.3) NG 9.87 (1.64) **MGL** SCTG −0.04 (0.3) XCM 0.42 (0.60) NG 0.72 (0.57) **BTT** SCTG 3.04 (0.61) XCM 2.1 (0.54) NG 2.11 (0.60)	Yes	Yes	facial pouch	12 mos.	SCTG Thin 5 thick 3 XCM Thin 4 thick 4 NG Thin 5 thick 3
**Abass et al, 2020** [38] **RCT**	14	22–45yr	CTG ADM	14 7 / 7	**KMW** SCTG 3.571(1.397) ADM 4.429 (1.058) **PES** SCTG 7.429 (0.787) ADM 7.600 (0.447) **Bleeding index** SCTG 0.929 (0.189) ADM 0.666 (0.193) **Probing depth** SCTG 2.903 (0.659) ADM 2.506 (0.317)	Yes / early provisionalization (2–3 weeks)	NR	A partial-thickness envelope.	12 mos.	SCTG / ADM Soft tissue biotype (< 2 mm) thin biotype

MBL marginal bone level, PES pink aesthetic score, KMW keratinized mucosa width, MGL mid-buccal mucosal level, BTT soft tissue thickness, IIPP immediate implant placement and provisionalization. NR not reported, SCTG subepithelial connective tissue graft, XCM xenogenic collagen matrix, ADM acellular dermal matrix, RCT randomized controlled trail, GBR guided bone regeneration.

### 2.5 Risk of bias assessment

Two authors (A. TA., MA. M) independently assessed the risk of bias in the included studies. Quality assessment of the risk of bias for all included studies was carried out using Cochrane collaboration’s tool. All studies were evaluated using the RCT checklist that involves random sequence generation, allocation concealment, blinding of outcome assessment, incomplete outcome data, selected reporting, and other biases. If all criteria were met, the study rated as a low risk of bias. If one or more key domains were unclear, the study considered an unclear risk of bias. Studies that did not meet one or more of these criteria were classified as having a high risk of bias. In case of disagreement, the consensus was reached by consultation with a third reviewer was performed (WLP.).

### 2.6 Certainty of the evidence

The Grading of Recommendations Assessment, Development and Evaluation (GRADE) approach of the meta-analysis was utilized to identify the certainty of effect estimates from the meta-analysis for all outcomes of interest. In the GRADE system, RCTs are rated as high-quality evidence but they may be downgraded due to limitations in one or more of the following domains: risk of bias, inconsistency, indirectness of evidence, imprecision, and publication bias [24]. The summary of confidence for the present evidence was estimated using RevMan [25].

### 2.7 Statistical analysis

The analysis was conducted to compare the effect of simultaneous soft tissue augmentation of different techniques on peri-implant tissue. All collected data in the current review were continuous data, and the weighted mean differences (MD) and 95% confidence interval (CI) were used to construct forest plots of selected studies. The heterogeneity across studies was assessed by the Cochrane Q test (χ^2^ test) and the I-squared index (*I*^2^). *I*^2^ = 0% to 25%, no heterogeneity; *I*^2^ = 25% to 50%, moderate heterogeneity; *I*^2^ = 50% to 75%, high heterogeneity; *I*^2^ = 75% to 100%, extreme heterogeneity [26]. When *I*^2^< 50%, we used the random effect model described by DerSimonian and Laird [27]. Otherwise, the data was regarded as homogeneous, and a fixed-effect model was used. The p-value of <0.05 was considered statistically significant. A Sub-group meta-analysis was conducted to evaluate the effect of different Variables on the outcomes of interest. The Cochrane Collaboration’s Review Manager Software (RevMan version 5.0) was utilized for data analysis.

### 2.8 Sensitivity analysis

Sensitivity analysis was performed to assess whether each individual study influenced the final results. This was performed by omitting one study at a time and calculating the pooled mean differences (MD) for the remaining studies.

## 3. Results

**Fig 1:**
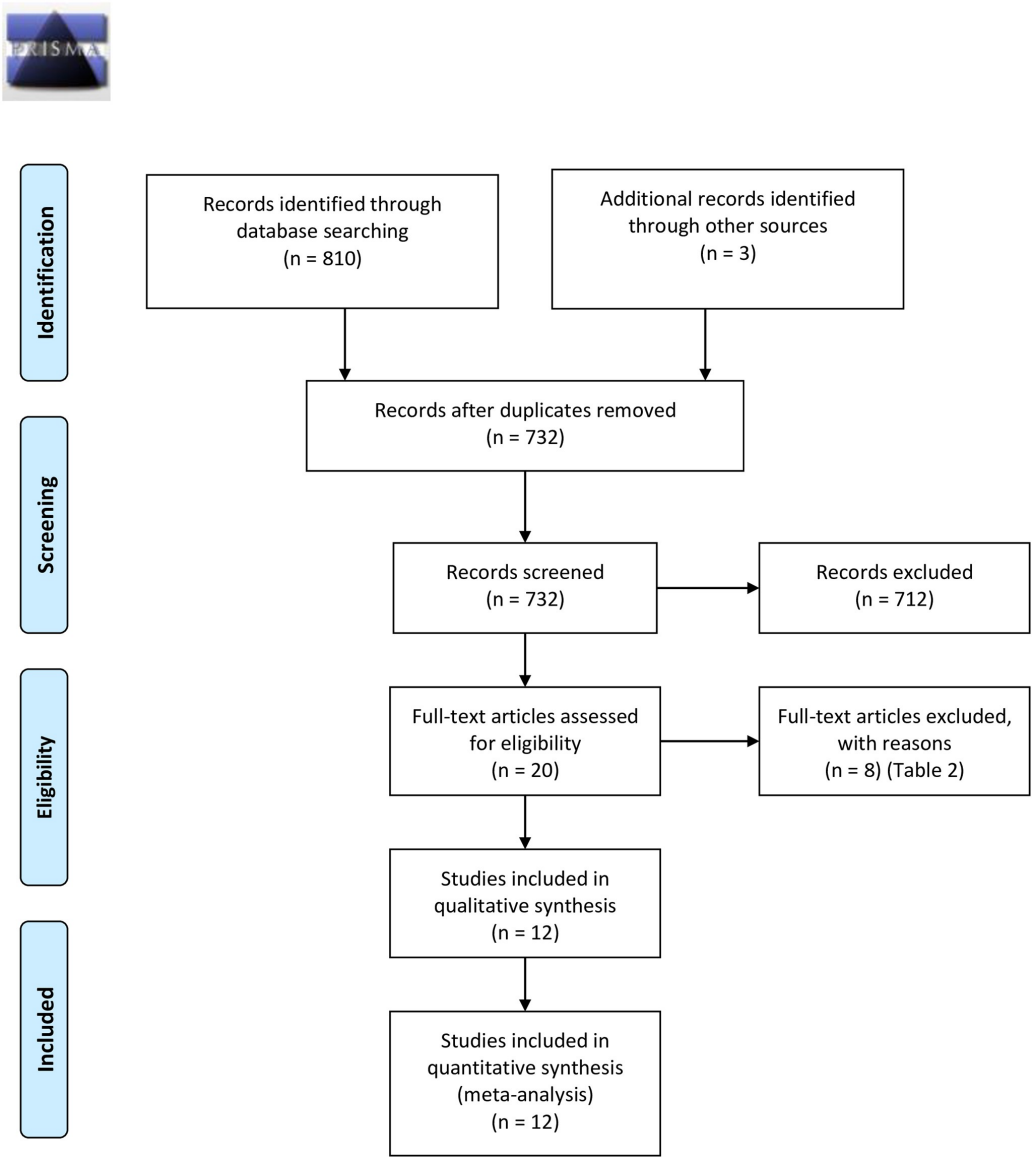
Study flow diagram.

The electronic and manual searches identified 813 articles. Seven hundred thirty-two records remained after duplicates were removed. The titles and abstracts of the remaining 732 articles were screened, and 712 were excluded due to being topic-off or non-English studies. Two researchers carefully read the full text of the remaining 20 studies for potential inclusion. Finally, 12 RCTs studies [5, 28–38] met the inclusion criteria and were included in our meta-analysis (Fig 1) (Table 1). The other 8 articles were excluded for reasons (Table 2). The follow-up ranged from four to 24 months. The minimum follow-up was reported to be 4 months in one study [30], whereas the maximum follow-up was reported to be 24 months [36] (Table 1).

**Table 2:** Excluded studies and reasons for exclusion.

Author(s)/ year	Title	Reason
**Lorenzo et al, 2011** [39]	Clinical efficacy of a xenogeneic collagen matrix in augmenting keratinized mucosa around implants: a randomized controlled prospective clinical trial.	Soft tissue augmentation was performed to treat gingival recession around an osseointegrated dental implant
**Rungcharassaeng et al. 2012** [40]	Immediate implant placement and provisionalization with and without a connective tissue graft: an analysis of facial gingival tissue thickness	No randomization was reported
**Bianchi and Sanfilippo, 2004** [41]	Single-tooth replacement by immediate implant and connective tissue graft: a 1–9-year clinical evaluation.	Data reported in percentage
**Zafiropoulos and John 2017** [42]	Use of Collagen Matrix for Augmentation of the Peri-implant Soft Tissue at the Time of Immediate Implant Placement.	Prospective non randomized
**Cairo et al. 2017** [43]	Xenogeneic collagen matrix versus connective tissue graft for buccal soft tissue augmentation at implant site. A randomized, controlled clinical trial	No simultaneous soft tissue augmentation was performed
**Thoma et al. 2016** [44]	Randomized controlled clinical study evaluating effectiveness and safety of a volume-stable collagen matrix compared to autogenous connective tissue grafts for soft tissue augmentation at implant sites.	Not simultaneous soft tissue augmentation.
**Ustaoglu et al, 2020** [45]	Titanium-Prepared Platelet-Rich Fibrin Versus Connective Tissue Graft on Peri-Implant Soft Tissue Thickening and Keratinized Mucosa Width: A Randomized, Controlled Trial	Short follow up (3 months)
**van Nimwegen et al, 2018** [46]	Immediate placement and provisionalization of implants in the aesthetic zone with or without a connective tissue graft: A 1-year randomized controlled trial and volumetric study	Duplicated article (Zuiderveld et al. 2018a)

### 3.1 Study characteristics

Twelve articles [5, 28–38] had 363 participants were included. The age of the patients ranged from 22 to 65 years. The follow-up ranged from 4 months to 2 years (Table 1). There were 11 patients lost from follow-up in four included studies [31, 34, 36, 38]. SCTG was compared with no graft along with IIP in four included studies [29, 35–37]. Two studies compared SCTG with no graft along with DIP [5, 33]. SCTG was compared with ADM in three included studies [30, 34, 38]. Three articles [28, 31, 32] compared SCTG with no augmentation plus guided bone regeneration (GBR). For assessment methods, various techniques have been used. BTT was measured in millimeters (mm) and was assessed using endodontic instruments in 2 studies [31, 36], ultrasonic device in one study [28]. For MGL, the outcome was measured in mm. Photographs with the periodontal probe in two studies [37, 46] cast were photographed with a millimeter grid in one study [36], and periodontal probe and cast in one study [35]. KT was assessed using the periodontal probe in four studies [30, 31, 34, 36, 38]. MBL was measured in mm and assessed using an intraoral radiograph in all studies. PES was assessed as described by Furhauser et al [47].

### 3.2 Quality assessment of the included studies

**Fig 2:**
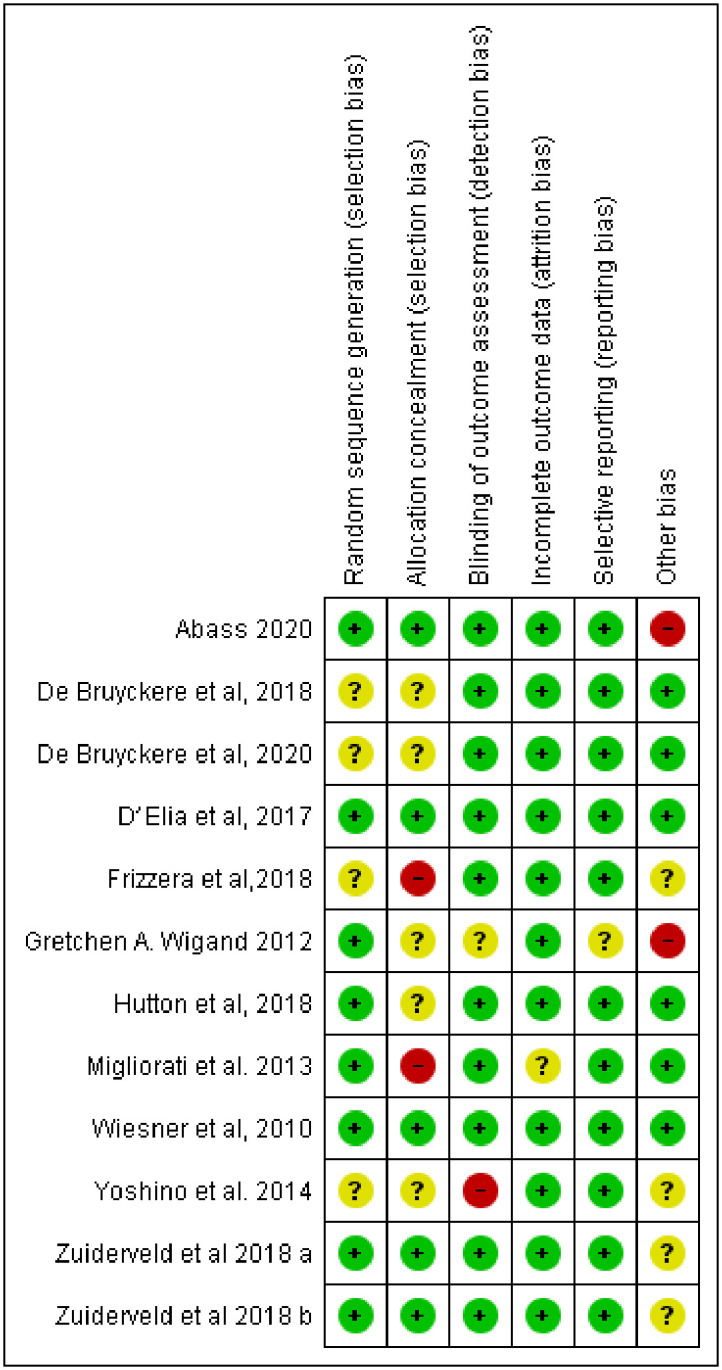
Result of risk of bias assessment of the included studies.

The full checklist (Cochrane Collaboration’s tool for assessing the risk of bias) was applied for the assessment of the included RCTs. Two trials [5, 31] were considered as a low risk of bias, Five articles [28, 30, 32, 33, 37] were rated as unclear risk, and five studies were rated as high risk of bias [29, 34–36, 38] (Fig 2).

### 3.3 Confidence of evidence

Based on the results of the GRADE assessment tools (S2 File), the quality of evidence for all analysis was rated as having a very low quality of evidence. The quality of evidence was downgraded because of limitations in the study design (risk of bias) and imprecision.

### 3.4 Results of individual outcome variables

#### 3.4.1 Soft tissue augmentation along with immediate implant placement (IIP)

*A- SCTG versus no graft plus IIP*. Four studies with a total of (n = 144) [29, 35–37] were included.

**Fig 3:**
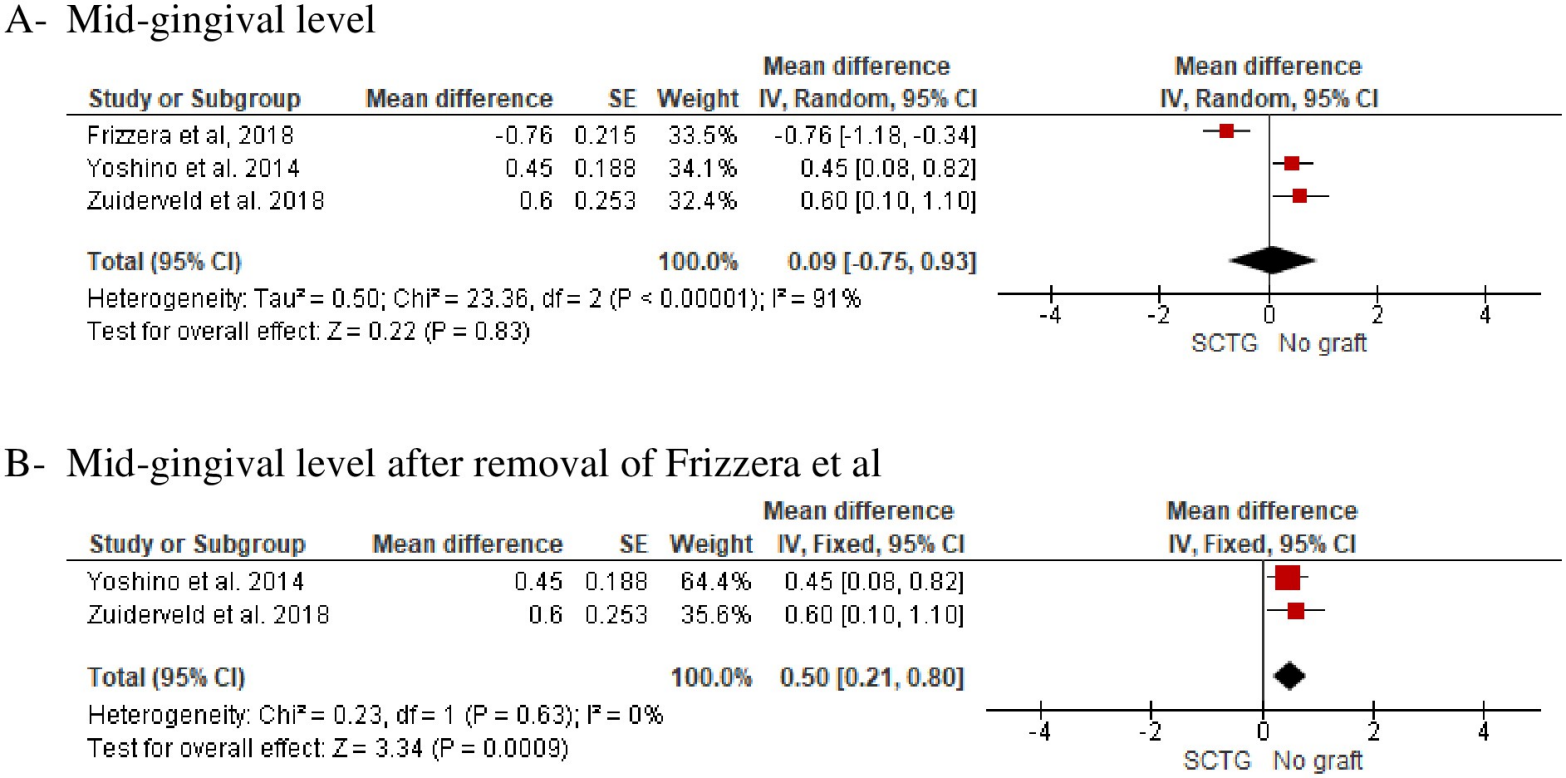
(A) Mid buccal recession, SCTG vs no graft along with IIP. (High heterogeneity was reported 91%). (B) Mid buccal recession, SCTG vs no graft along with IIP. (After removal of the study conducted by Frizzera et al, the heterogeneity changed to 0%).

**Fig 4:**
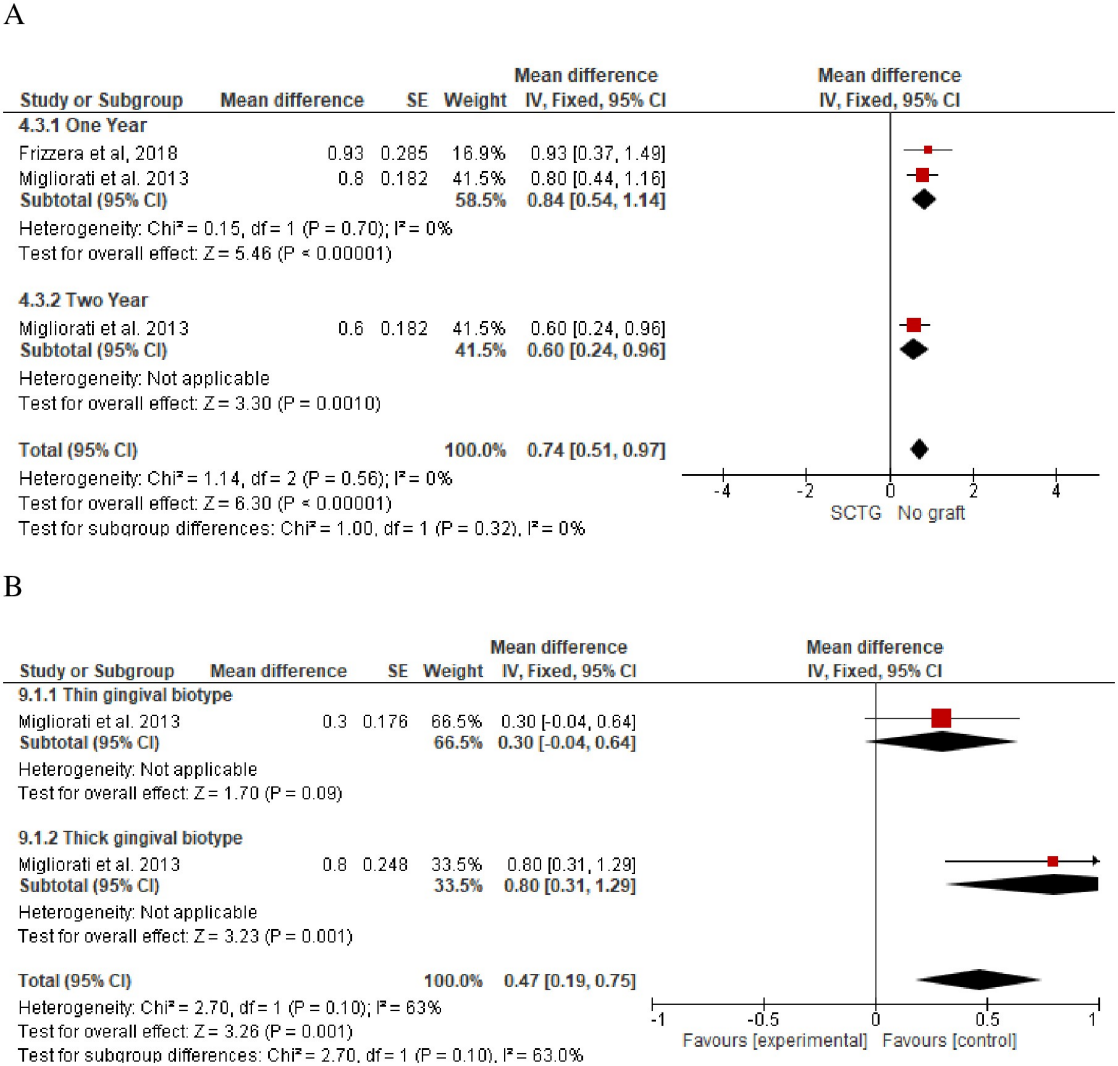
Buccal tissue thickness, SCTG vs no graft along with IIP. A) A subgroup meta-analysis with one- and two-years follow-up. B) A subgroup meta-analysis with thin or thick gingival biotypes.

**Fig 5:**
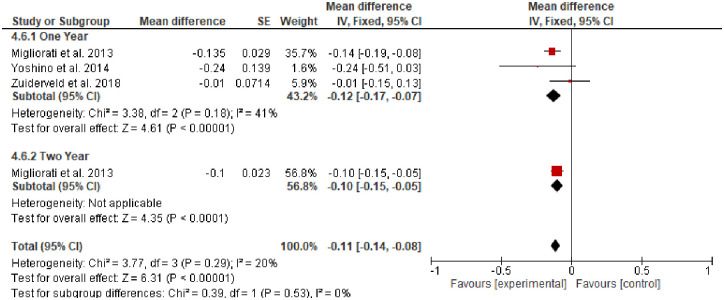
Marginal bone loss, SCTG vs no graft along with IIP.

**Fig 6:**
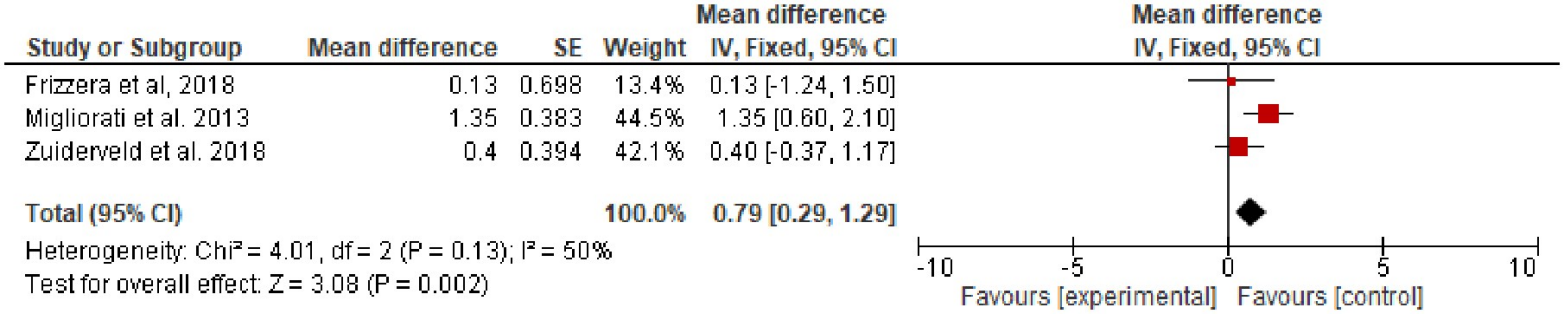
Pink aesthetic score SCTG vs no graft along with IIP.

- **Mid-buccal gingival level (MGL)** was evaluated in three studies [29, 35, 37] with a total of 96 participants (SCTG = 48, No graft = 48) and follow-up of 12 to 24 months. SCTG showed a statistically insignificant difference in the MGL compared to no graft (Random; MD, 0.09; 95% CI, -0.95, 0.93, *P = 0*.*83*). However, a high heterogeneity of about *91*% was observed and after removal of Frizzera et al’s study, SCTG (n = 40) showed a statistical significant difference in MGL compared to no graft (n = 40) and 0% heterogenity was observed (Fixed; MD, 0.50; 95% CI, 0.21, 0.80, *P = 0*.*0009*, *I*^*2*^ = *0*). (Fig 3B).
- **Buccal tissue thickness (BTT)** was evaluated in two included studies with a total of 64 participants [29, 36]. Migliorati et al [36] tested SCTG versus no graft at 12 and 24-months of follow-up. Frizzera et al [29] evaluated SCTG versus no graft at 12 months. Meta-analysis of the two included studies showed a statistically significant increase in the BTT in favor of SCTG (Fixed, MD, 0.84; 95% CI, 0.54, 1.14, *P = 0*.*001*, *I2 = 0%*). There was a reduction in the BTT of about 2.4 mm after two years of follow-up compared to one year (Fig 4A). One study [36] conducted a subgroup analysis regarding gingival thickness biotype (thin or thick) and a subgroup meta-analysis in the group of patients with thin gingival biotype along with SCTG showed a statistically insignificant increase of about 0.3 mm in BTT compared to patients with no augmentation and thin gingival biotype (Fixed; MD, 0.3; 95% CI, -0.04, 0.64, *P = 0*.*09*). However, a meta-analysis of thick gingival biotype who received SCTG showed a statistically significant increase in the BTT of about 0.8 mm compared with no graft and thick gingival biotype (Fixed; MD, 0.8; 95% CI, 0.31, 1.29, *P = 0*.*001*) (Fig 4B).
- **Marginal bone loss** was reported in three studies with a total of 108 participants [35–37]. SCTG showed a statistically significant less marginal bone loss of about 0.12 and 0.1 after one and two years follow-up (Fixed; MD, − 0.12; 95% CI, -0.17 –-0.07, *P = 0*.*001*) and (Fixed; MD, -0.1; 95% CI, -0.15, -0.05, *P = 0*.*001*) respectively (Fig 5).
- **Pink aesthetic score** was reported in three studies with 124 participants [29, 36, 37] along with IIP. SCTG showed a statistically significant improvement in the pink aesthetic score compared to no graft (Fixed; MD, 0.79; 95% CI, 0.29, 1.29, *P = 0*.*002*) (Fig 6).

*B- SCTG versus other augmentation techniques (XCM or ADM) plus IIP*.

- Frizzera et al [29] compared SCTG with CM regarding MR, BTT, and PES. The differences in MGL and BTT after 6 and 12 months were statistically significant favoring SCTG. The differences in MGL after 6 and 12 months were (Mean (SD)-0.14 (0.75) versus 0.14(0.37) and 0.04(0.3) versus 0.42(0.60)) respectively, whereas the differences in BTT were (2.82 mm ± 0.40 vs 2.05 mm ± 0.41; P < .001) and (3.04 mm ± 0.61 vs 2.1 mm ± 0.54; P < .001) respectively. This study was considered as a high risk of bias. No statistically significant differences were observed regarding PES (P>0.05) (Table 1).
- Abbas [38] tested SCTG versus ADM in conjunction with IIP. After 12 months, the differences were statistically insignificant differences regarding KMW (P = 0.22) and PES (P = 0.33).

#### 3.4.2 Soft tissue augmentation along with DIP

*A-SCTG versus no graft plus DIP*.

- One study (n = 60) [33] with 1-year follow-up compared two types of soft tissue augmentation (SCTG and XCM) with no graft along with DIP in the preserved socket. After 12 months of follow-up, there were no statistically significant changes in the MGL, PES, marginal bone level, and clinical peri-implant (P>0.05).
- Wiesner et al [5] performed simultaneous soft tissue augmentation using SCTG in conjunction with DIP. After 12 months follow-up, there was a statistically significant change in the BTT (mean change 1.3 mm, P = 0.001), MBL (mean change 0.79 mm, P<0.05), and PES (P = 0.001) favoring SCTG.

**Fig 7:**
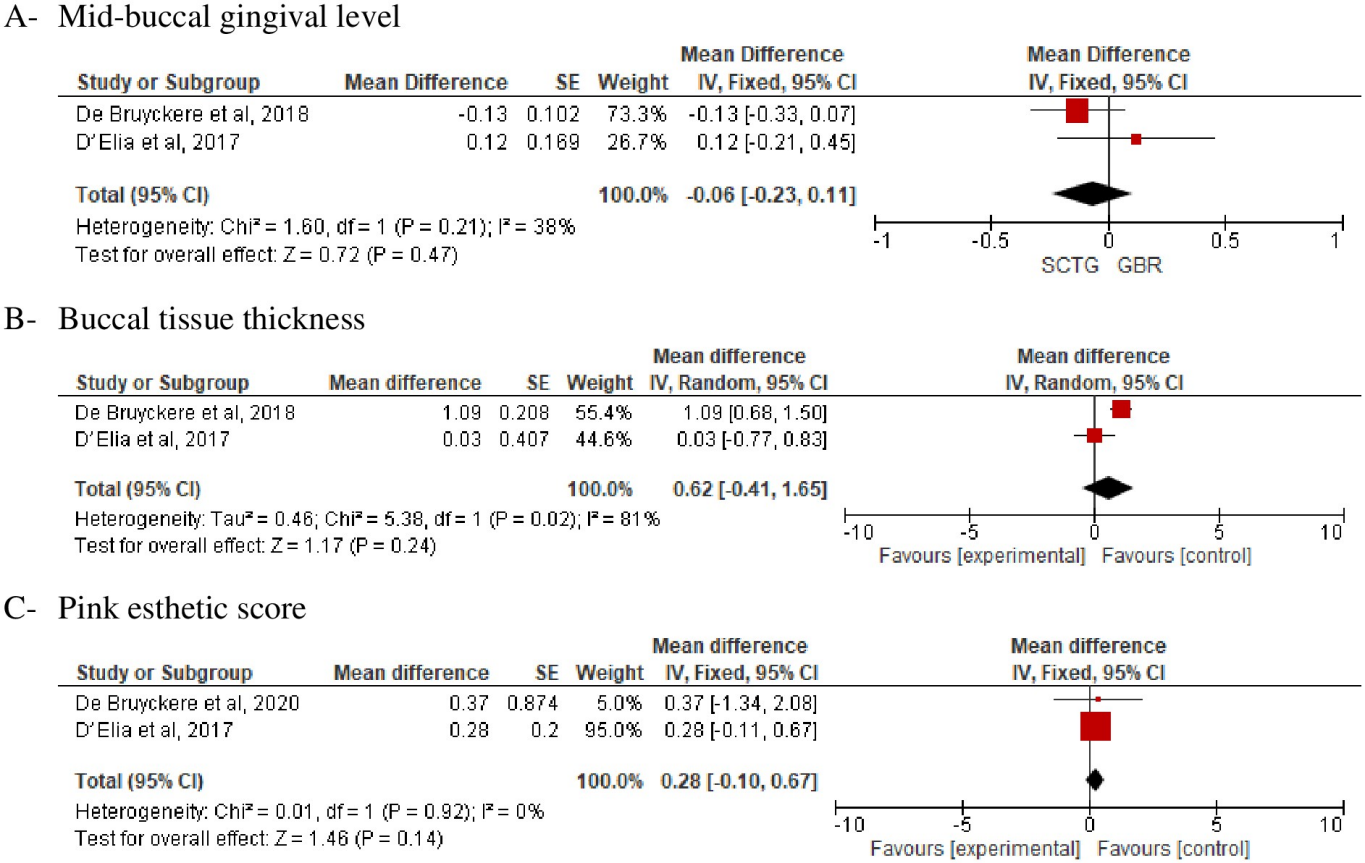
SCTG vs no augmentation plus GBR along with DIP. A) Mid buccal recession, B) Buccal tissue thickness, C) Pink aesthetic score.

**Fig 8:**
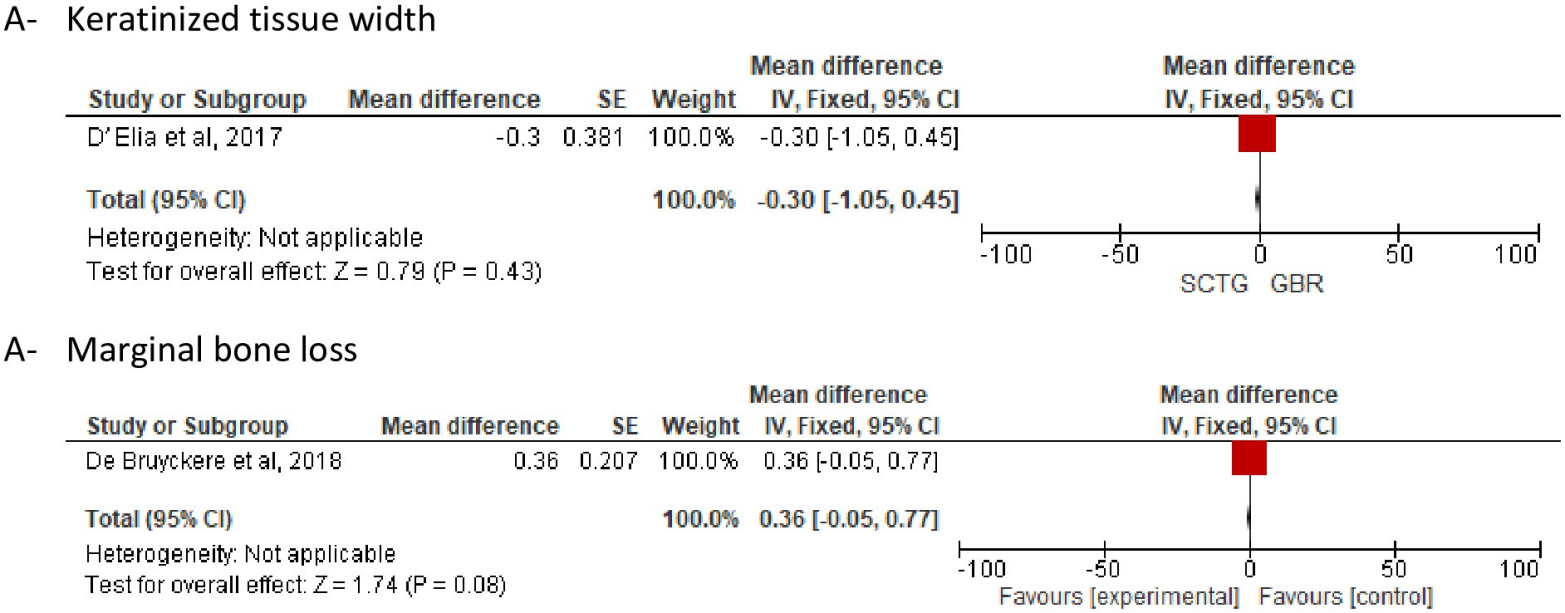
SCTG vs GBR along with DIP. A) Keratinized tissue width, B) Marginal bone loss.

*B-SCTG versus No soft tissue grafting plus GBR with DIP*. Three articles with a total sample size of n = 74 patients compared SCTG with no augmentation plus GBR [28, 31, 32]. Two studies of the same trial and same participants but with different outcomes were conducted by De Bruyckere et al [28, 32]. The three studies were included in the evaluation of the following outcomes:

Two studies [28, 31] compared SCTG with no augmentation plus GBR in regarding MGL and BTT, and a statistically insignificant difference was observed (Fixed; MD, -0.06; 95% CI, -0.23–0.11, *P* = 0.47) (Random; MD, 0.62; 95% CI, -0.41–1.65, *P = 0*.*24*) (Fig 7A and 7B). Also, no statistically significant difference was observed regarding MBL and KMW then comparing SCTG with no augmentation plus GBR (Fig 8).

Pink aesthetic score was evaluated in two studies [31, 32], there was no statistically significant difference between SCTG and GBR (Fixed; MD, 0.28; 95% CI, -0.10–0.64, *P = 0*.*14*) (Fig 7C).

*C. SCTG versus other augmentation techniques (CM or ADM) plus DIP*.

- Two studies compared SCTG with ADM along with DIP, a total of 20 patients with 4 months follow-up were included in the study performed by Hutton et al [30]. Four months postoperatively, no statistically significant differences in terms of BTT (MD:0.71, P = 0.18) and KMW (MD: 0.6, P = 0.19) were observed between ADM and SCTG. Another study compared SCTG with ADM with a total of 24 patients and 12 months follow-up [34] and showed a statistically insignificant increase in KMW of about 0.10 mm. One study [34] evaluated pink aesthetic score, and there was no statistical difference between SCTG and ADM (P = 0.25).

## 4. Discussion

Based on the results of the GRADE assessment, there is a very low quality of evidence showing that simultaneous soft tissue augmentation around immediate or delayed dental implant placement results in an improvement in the quality and the quality of the peri-implant tissue. The present study showed that soft tissue augmentation is a beneficial procedure to prevent a mid-facial recession, increase BTT, and reduce MBL, and this was regardless of the timing of implant placement protocol used. SCTG was compared with no augmentation in conjunction with IIP in 2 included studies [35, 37] with a total of 80 patients; there was a statistically significant more coronally placed MGL favoring SCTG (Fixed; MD, 0.50; 95% CI, 0.21, 0.80, *P = 0*.*0009*). This result is in line with some clinical studies in that simultaneous soft tissue grafting under gingival mucosa resulted in an increase in the height and thickness of the peri-implant tissues [40, 48], and this surgical option can be considered in cases with non-salvageable teeth showing gingival recession, in cases of absence of attached gingiva, and to conceal underlying implant restorative materials [37, 46, 49].

The result obtained from the present systemic review and meta-analysis support that soft tissue augmentation using SCTG significantly improve BTT around dental implant regardless of whether immediate or delayed placement protocol was employed. The gains in BTT after one year of follow-up were 0.84 mm in the studies that performed simultaneous SCTG augmentation along with IIP and 1.3 mm in the study performed SCTG along with DIP. However, these results should be interpreted with caution because of high heterogeneity among studies that evaluated SCTG along with IIP, and only a single study evaluated SCTG along with DIP.

One important factor that has been considered as a prognostic factor for the esthetic outcome is the gingival biotype [50, 51]. It has been reported that gingival thickness at the crest plays a crucial role in marginal bone stability around the implant. Further, it has been reported that less mid-buccal recession occurs in the thick gingival biotype group compared with the thin group [52]. Kan et al demonstrated that sites with a thick gingival biotype exhibited significantly smaller changes in facial gingival level than sites with a thin gingival biotype [53]. Farina and Zaffe [54] concluded that soft tissue augmentation under thin gingival biotype using ADM increases gingival thickness more than that in the thick gingival biotype, whereas a decrement was found in control sites with no graft used. Our finding is consistent with the study conducted by Speroni et al [55] in that the thick gingival biotype along with SCTG showed a statistically significant increase in BTT of about 0.8 mm compared with thin gingival biotype which showed a statistically insignificant change of about 0.3 mm. However, this result was obtained from a single study with a very low quality of evidence [36].

A subgroup meta-analysis of the change in the mucosal thickness after one and two years showed a mean difference of about 0.84 mm and 0.60 mm respectively when SCTG compared with no graft along with IIP. The mean loss of the BTT between 1year and 2 years was minimal about 0.24 mm. This was in line with the study conducted by Sanz-Martín et al [56] who reported a mean reduction of about 0.3 mm in the buccal tissue contours between 6 months and 1 year. Interestingly, some studies reported a considerable increase in the soft tissue thickness after immediate implantation placement even if no soft tissue augmentation was used [40, 57].

GBR could also be considered to play a role in increasing the stability of peri-implant soft tissue and preventing marginal tissue shrinkage. It has been reported that marginal gingival change may occur after immediate implant placement particularly in the esthetic zone [58]. Therefore, it has been suggested to fill the gap between the implant and buccal bone plate with a bone graft to reduces bone resorption [59]. Unfortunately, no study in the current meta-analysis tested the effect of SCTG versus GBR along with IIP. Instead, two trials that compared the effect of simultaneous soft tissue augmentation using SCTG with GBR [28, 31] along with delayed implant placement were included. Surprisingly, the results seem that GBR produced a comparable effect to the SCTG in improving BTT, MGL, and PES P = 0.24, 0.47, and 0.14 respectively.

Comparing SCTG versus XCM or ADM along with DIP showed a comparable effect in improving the quality and quantity of the peri-implant tissue. Lorenzo et al. [39] reported that no statistical difference was observed between SCTG and XCM regarding the buccal recession (*P = 0*.*667*) and this was in line with our findings. Huber et al. [60] and Thoma et al. [17] showed no statistically significant difference in buccal tissue thickness when SCTG compared with XCM. Alternatively, Cairo et al. [43] showed that a significant increase in BTT in the SCTG group when compared with the XCM group. However, these studies [43, 60] were excluded from the current review because soft tissue augmentation was not performed simultaneously at the time of implant placement (Table 2). Comparing SCTG with ADM concerning MGL, BTT, MBL, and PES showed no statistically significant difference P>0.05. This finding was in line with the studies conducted by Liu et al. [61] which was excluded from the current meta-analysis because the article was written in the Chinese language. Also, this result was in line with the studies that compared the effect of SCTG with ADM in the treatment of gingival recession around natural teeth [62, 63].

We notice several limitations in the current meta-analysis that should be declared. First, most of the included studies were assessed as having a high risk of bias. Second, only RCTs were assessed which result in a limitation in the number of included studies; therefore, considering the inclusion of both RCT and non-RCT studies in the future meta-analysis would be beneficial to ensure that all relevant information will be tested. Third, the small sample size in the included studies. Fourth, high heterogeneity in some analyses due to the difference in study design, recruitment of the participants, and methods used for the assessment of outcomes. Fifth, the non-English studies in the current review were excluded. Finally, several cofounders that may affect on the outcomes of interest were not evaluated by most of the included studies, such as follow-up time, site of SCTG harvest (from the palate or maxillary tuberosity), gingival biotype, implant diameter, implant system, implant surface, implant design, type of abutment used, using bone graft or not, and whether immediate provisionalization was used or not. However; for an optimum comparison of different peri-implant tissue augmentation surgeries with the least bias, homogeneous sample of participants with the same implant placement techniques (IIP and DIP), implant insertion site, augmentation techniques (soft and/or hard augmentation), follow up times (>3 months follow-up period) is recommended. All the aforementioned limitations preventing us from drawing a defective conclusion regarding the effect of simultaneous soft tissue augmentation around immediate or delayed dental implant placement on the peri-implant health and aesthetic. Therefore, the results of the current meta-analysis should be interpreted with caution, and further RCTs with a large sample size, longer follow-up period, and clearer design that compares the SCTG and no graft or other soft tissue substitutes are required.

## 5. Conclusion

Within the limitations of the current meta-analysis, it seems that:

1. Simultaneous soft tissue augmentation using SCTG at the time of immediate implant placement improves BTT and PES, prevents mid-buccal recession, and reduced MBL compared with NG.
2. SCTG compared with GBR along with DIP showed a statistically insignificant difference concerning MGL, BTT, and PES.
3. ADM and CM in conjunction with DIP produce a comparable effect to SCTG in improving periimplant quality and quantity.

However, this conclusion should be interpreted with caution due to the very low quality of evidence for all analyses. Therefore, further, well-designed RCTs with larger sample sizes and long follow-up times are still needed.

## Supporting information

S1 Checklist PRISMA 2009 checklist in this meta-analysis. (DOCX)

S1 File Search strategies in different databases. (DOCX)

S2 File Certainty of evidence (GRADE). (DOCX)
