# Supplementary material for: Does simultaneous soft tissue augmentation around immediate or delayed dental implant placement using sub-epithelial connective tissue graft provide better outcomes compared to other treatment options? A systematic review and meta-analysis
Source: PLoS One. 2022 Feb 10;17(2):e0261513. doi: 10.1371/journal.pone.0261513 (PMC8830641; doi:10.1371/journal.pone.0261513)
Supplement: S1 File — (DOCX) [file pone.0261513.s002.docx]

| Data base | Keywords | Yielded studies |
| --- | --- | --- |
| PubMed  10-May-2021 | ((immediate implant [Title/Abstract]) OR (immediate implant placement [Title/Abstract]) OR (early implant placement [Title/Abstract])) OR (delayed implant placement [Title/Abstract])) AND ((soft tissue graft [Title/Abstract]) OR (sub-epithelial connective tissue graft [Title/Abstract]) OR (connective tissue [Title/Abstract]) OR (soft tissue augmentation [Title/Abstract]) OR (soft tissue transplantation [Title/Abstract]) OR Xenograft [Title/Abstract])) OR heterografts [Title/Abstract])) OR collagen matrix [Title/Abstract])) OR mucograft [Title/Abstract])) OR Acellular dermal matrix [Title/Abstract])) OR acellular dermis [Title/Abstract])) AND ((attached gingiva[Title/Abstract]) OR (buccal soft tissue thickness [Title/Abstract]) OR (keratinized mucosa[Title/Abstract]) OR (soft tissue margin[Title/Abstract]) OR (pocket probing depth [Title/Abstract]) OR (esthetic [Title/Abstract])). | 227 |
| **Embase**  10-May-2021 | ((('tooth implant':ab,ti OR 'delayed dental implant placement':ab,ti OR 'immediate delayed dental implant placement':ab,ti) AND 'soft tissue augmentation':ab,ti OR 'acellular dermal matrix':ab,ti OR 'xenogeneic collagen matrix':ab,ti OR 'connective tissue':ab,ti) AND 'alveolar bone loss':ab,ti OR 'gingival recession':ab,ti OR 'keratinized tissue width':ab,ti OR 'pink esthetic score':ab,ti) AND 'randomized controlled trial':ab,ti | 38 |
| Cochrane Library  10-May-2021 | #1 MeSH descriptor: [Dental Implants] explode all trees  #2 (delayed dental implant loading):ti,ab,kw  #3 MeSH descriptor: [Immediate Dental Implant Loading] explode all trees  #4 #1 OR #2 OR #3  #5 (soft tissue augmentation):ti,ab,kw  #6 MeSH descriptor: [Acellular Dermis] explode all trees  #7 MeSH descriptor: [Heterografts] explode all trees  #8 (xenogeneic collagen matrix):ti,ab,kw  #9 (connective tissue graft):ti,ab,kw  #10 #5 OR #6 OR #7 OR #8 OR #9  #11 MeSH descriptor: [Alveolar Bone Loss] explode all trees  #12 MeSH descriptor: [Gingival Recession] explode all trees  #13 (keratinized tissue width):ti,ab,kw  #14 (pink esthetic score):ti,ab,kw  #15 #11 OR #12 OR #13 OR #14  #16 #4 AND #10 AND #15 | 48 |
| Google Scholar  10-May-2021 | Dental implant OR Xenograft OR Acellular dermal matrix OR Alveolar bone loss OR Buccal tissue thickness OR Mid buccal recession OR Pink aesthetic score "Connective tissue graft" | 497 |

Supplemental File **S2 Search strategies in different databases**
