# Supplementary material for: Does simultaneous soft tissue augmentation around immediate or delayed dental implant placement using sub-epithelial connective tissue graft provide better outcomes compared to other treatment options? A systematic review and meta-analysis
Source: PLoS One. 2022 Feb 10;17(2):e0261513. doi: 10.1371/journal.pone.0261513 (PMC8830641; doi:10.1371/journal.pone.0261513)
Supplement: S2 File — (DOCX) [file pone.0261513.s003.docx]

**Supplemental file S2:** Certainty of evidence (GRADE), simultaneous soft tissue augmentation around immediate or delayed dental implant placement using SCTG versus other treatment modalities.

| **Certainty assessment** | | | | | | | **№ of patients** | | **Effect** | | **Certainty** | **Importance** |
| --- | --- | --- | --- | --- | --- | --- | --- | --- | --- | --- | --- | --- |
| **№ of studies** | **Study design** | **Risk of bias** | **Inconsistency** | **Indirectness** | **Imprecision** | **Other considerations** | **SCTG** | **No graft** | **Relative (95% CI)** | **Absolute (95% CI)** |  |  |
| **Mid-gingival level (MGL) - SCTG vs No graft (follow up: 12 months; assessed with: Photograph at different follow-up interval)** | | | | | | | | | | | | |
| 2 | randomized trials | Very serious ^a,b^ | not serious | not serious | serious ^b^ | none | 40 | 40 | - | MD 0.50 (0.21 to 0.80) | ⨁◯◯◯ VERY LOW |  |
| **Marginal bone loss (MBL), SCTG vs No graft (follow-up: 12 months)** | | | | | | | | | | | | |
| 3 | randomized trials | very serious ^a,b^ | not serious | not serious | serious ^b^ | none | 64 | 64 | - | MD -**0.12**  (-0.17 to -0.07) | ⨁◯◯◯ VERY LOW |  |
| **Marginal bone loss (MBL), SCTG vs No graft (follow-up: 24 months)** | | | | | | | | | | | | |
| 1 | randomized trials | Very serious ^a,b^ | not serious | not serious | serious ^b^ | none | 24 | 24 | - | MD -0.11  (0.14 to 0.08) | ⨁◯◯◯ VERY LOW |  |
| **Buccal tissue thickness (BTT), SCTG vs No graft (follow-up: 12 months)** | | | | | | | | | | | | |
| 2 | randomized trials | Very serious ^a,b^ | not serious | not serious | serious ^b^ | none | 34 | 34 | - | MD **0.84** (0.54 to 1.14) | ⨁◯◯◯ VERY LOW |  |
| **Buccal tissue thickness (BTT), SCTG vs No graft (follow-up: 24 months)** | | | | | | | | | | | | |
| 1 | randomized trials | randomized trials | Very serious ^a,b^ | not serious | not serious | serious ^b^ | none | 24 | - | MD **0.60** (0.26 to 0.96) | ⨁◯◯◯ VERY LOW |  |
| **Pink esthetics score, SCTG vs No graft, follow up: 12 months** | | | | | | | | | | | | |
| 3 | randomized trials | very serious ^a,b^ | not serious | not serious | serious ^b^ | none | 64 | 64 | - | MD **0.79** (0.29 to 1.29) | ⨁◯◯◯ VERY LOW |  |
|  | | | | | | | | | | | | |

#### Explanations

a. Drop out

b. small sample size

c. cross null

d. Risk of bias

e. long CI

| **Certainty assessment** | | | | | | | **№ of patients** | | **Effect** | | **Certainty** | **Importance** |
| --- | --- | --- | --- | --- | --- | --- | --- | --- | --- | --- | --- | --- |
| **№ of studies** | **Study design** | **Risk of bias** | **Inconsistency** | **Indirectness** | **Imprecision** | **Other considerations** | **SCTG** | **No graft** | **Relative (95% CI)** | **Absolute (95% CI)** |  |  |
| **Mid-gingival level (MGL) - SCTG vs GBR (follow up: 12 months)** | | | | | | | | | | | | |
| 2 | randomized trials | Very serious ^a,b^ | not serious | not serious | serious ^b^ | none | 37 | 37 | - | MD -0.06 (-0.23 to 0.11) | ⨁◯◯◯ VERY LOW |  |
| **Buccal tissue thickness (BTT), SCTG vs GBR (follow-up: 12 months)** | | | | | | | | | | | | |
| 2 | randomized trials | Very serious ^a,b^ | not serious | not serious | serious ^b^ | none | 37 | 37 | - | MD -**0.62**  (-0.41 to -1.65) | ⨁◯◯◯ VERY LOW |  |
|  | | | | | | | | | | | | |
| **Pink esthetics score, SCTG vs No graft, follow up: 12 months** | | | | | | | | | | | | |
| 2 | randomized trials | Very serious ^a,b^ | not serious | not serious | serious ^b^ | none | 37 | 37 | - | MD 0.28  (-0.11 to 0.67) | ⨁◯◯◯ VERY LOW |  |
| **Keratinized tissue width, SCTG vs GBR, follow up: 12 months** | | | | | | | | | | | | |
| 1 | randomized trials | Very serious ^a,b^ | not serious | not serious | serious ^b^ | none | 16 | 16 | - | MD- 0.30 (-1.05 to 0.45) | ⨁◯◯◯ VERY LOW |  |

| **Marginal bone loss, SCTG vs GBR, follow up: 12 months** | | | | | | | | | | | | |
| --- | --- | --- | --- | --- | --- | --- | --- | --- | --- | --- | --- | --- |
| 1 | randomized trials | Very serious ^a,b^ | not serious | not serious | serious ^b^ | none | 21 | 21 | - | MD 0.36 (-0.05 to 0.77) | ⨁◯◯◯ VERY LOW |  |

**CI:** Confidence interval; **SMD:** Standardised mean difference

**Explanations**

a. drop out

b. small sample size

c. risk of bais

d. CI overlapping with null

e. statistically difference between direct and indirect
